# Salsoline derivatives, genistein, semisynthetic derivative of kojic acid, and naringenin as inhibitors of A42R profilin-like protein of monkeypox virus: *in silico* studies

**DOI:** 10.3389/fchem.2024.1445606

**Published:** 2024-09-10

**Authors:** Mohamed Chebaibi, Mohammed Bourhia, Fatima ez-zahra Amrati, Meryem Slighoua, Ibrahim Mssillou, Mourad A. M. Aboul-Soud, Asaad Khalid, Rym Hassani, Dalila Bousta, Sanae Achour, Rachid Benhida, Rachid Daoud

**Affiliations:** ^1^ Ministry of Health and Social Protection, Higher Institute of Nursing Professions and Health Techniques, Fez, Morocco; ^2^ Department of Chemistry and Biochemistry, Faculty of Medicine and Pharmacy, Ibn Zohr University, Laayoune, Morocco; ^3^ Laboratory of Biotechnology, Environment, Agri-Food, and Health (LBEAS), Faculty of Sciences, University Sidi-Mohamed-Ben-Abdellah (USMBA), Fez, Morocco; ^4^ Laboratory of Natural Substances, Pharmacology, Environment, Modeling, Health and Quality of Life (SNAMOPEQ), Faculty of Sciences Dhar El Mahraz, Sidi Mohamed Ben Abdellah University, Fez, Morocco; ^5^ Department of Clinical Laboratory Sciences, College of Applied Medical Sciences, King Saud University, Riyadh, Saudi Arabia; ^6^ Health Research Center, Jazan University, Jazan, Saudi Arabia; ^7^ Environment and Nature Research Centre, Jazan University, Jazan, Saudi Arabia; ^8^ National Agency of Medicinal and Aromatic Plants Tounate, Taounate, Morocco; ^9^ Biomedical and Translational Research Laboratory, Faculty of Medicine and Pharmacy of Fez, Sidi Mohamed Ben Abdellah University, Fez, Morocco; ^10^ Chemical and Biochemical Sciences-Green Processing Engineering, Mohammed VI Polytechnic University, Ben Guerir, Morocco

**Keywords:** monkeypox virus, natural compounds, virtual screening, molecular dynamics simulation, tecovirimat

## Abstract

Monkeypox virus (MPV) infection has developed into a re-emerging disease, and despite the potential of tecovirimat and cidofovir drugs, there is currently no conclusive treatment. The treatment’s effectiveness and cost challenges motivate us to use *In Silico* approaches to seek natural compounds as candidate antiviral inhibitors. Using Maestro 11.5 in Schrodinger suite 2018, available natural molecules with validated chemical structures collected from Eximed Laboratory were subjected to molecular docking and ADMET analysis against the highly conserved A42R Profilin-like Protein of Monkeypox Virus Zaire-96-I-16 (PDB: 4QWO) with resolution of 1.52 Å solved 3D structure. Compared to the FDA-approved Tecovirimat, molecular docking revealed that Salsoline derivatives, Genistein, Semisynthetic derivative of kojic acid, and Naringenin had strengthened affinity (−8.9 to −10 kcal/mol) to 4QWO, and the molecular dynamic’s simulation confirmed their high binding stability. In support of these results, the hydrogen bond analysis indicated that the Salsoline derivative had the most robust interaction with the binding pockets of 4QWO among the four molecules. Moreover, the comparative free energy analyses using MM-PBSA revealed an average binding free energy of the complexes of Salsoline derivative, Genistein, Semisynthetic derivative of kojic acid, Naringenin, of −106.418, −46.808, −50.770, and −63.319 kJ/mol, respectively which are lower than −33.855 kJ/mol of the Tecovirimat complex. Interestingly, these results and the ADMET predictions suggest that the four compounds are promising inhibitors of 4QWO, which agrees with previous results showing their antiviral activities against other viruses.

## Introduction

Monkeypox virus (MPV) infection has become persistent (Marennikova et al., 1972). MPV was initially identified in human populations in 1970 in the Democratic Republic of the Congo, and subsequently, the disease’s occurrence remained limited to this region (Marennikova et al., 1972). Later, MPV extended its presence to several African countries (Bunge et al., 2022; Meyer et al., 2002). In 1996 and 1997, the Democratic Republic of the Congo experienced outbreaks with low case fatality rates and attack rates higher than the norm. Since 2017, Nigeria has recorded over 500 suspected cases, with more than 200 confirmed cases and a case-fatality ratio exceeding 3% (Heymann et al., 1998). The virus’s first appearance outside Africa was recorded in 2003 in the USA (Reynolds et al., 2006). From 2018 to 2022, more than 40 countries, including the United Kingdom, Spain, Portugal, and Germany, identified a hundred infections (Do; Kumar et al., 2022). Historically, the case fatality rate for monkeypox in the general population varied from 0% to 11% and was more significant in young children (Nuzzo et al., 2022).

MPV is a double-enveloped DNA virus classified within the Poxviruses genus, part of the Poxvirus family (Kugelman et al., 2014). Two distinct groups of MPV have been identified: the Congo Basin group (Central African group) and the West African group. The Congo Basin group is thought to be more transmissible and has a history of causing more severe illnesses. (Likos et al., 2005). Based on the recent genomic sequencing data, the DNA sequences of the MPV strains found in Europe, specifically in Portugal, align with the West African clade, which implies a potentially less severe manifestation of the disease (Velavan and Meyer, 2022). In 2022, potential viral mutations are suggested to be responsible for the rapid transmission of monkeypox outbreak (Kumar et al., 2022) through direct contact with the blood, bodily fluids, skin lesions, or mucous secretions of sick animals, and animal-to-human infection (Angelo et al., 2019). Although several animals, such as rope squirrels and tree squirrels, have displayed indications of MPV infection (Tesh et al., 2004), the natural reservoir for MPV has not yet been found (Arita and Henderson, 1976) (World health organization, 2022a). MPV incubation (World health organization, 2022b; Kabuga and El Zowalaty, 2019) covers two stages: i) the invasion period (0–5 days long), which is characterized by fever, severe headache, enlarged lymph nodes, painful back and muscle pain, and significant weakness, and ii) the rash phase which frequently begins 1–3 days following the fever symptoms (McCollum and Damon, 2014). Several thousand lesions or sores merge in severe cases, leading to skin shedding (World health organization, 2022a; Kabuga and El Zowalaty, 2019; McCollum and Damon, 2014). A range of complications are known to be associated with MPV, including secondary infections, pneumonia, sepsis, encephalitis, and corneal infections leading to vision loss.

Several studies have revealed that smallpox immunization prevents 85% of the MPV infections. Therefore, a novel two-dose vaccine based on the attenuated modified vaccinia virus (Ankara strain) was authorized in 2019; however, this treatment option still confronts limited supplies and cost challenges (World health organization, 2022b; Gruber, 2022). The European Medicines Agency 2022 authorized an antiviral agent known as Tecovirimat (TPOXX^®^) to treat monkeypox, initially developed to treat smallpox but is not yet widely available (Sherwat et al., 2022; Rizk et al., 2022). Tecovirimat, an antiviral agent thwarting orthopoxvirus activity and preventing the production of infective virions, gained FDA approval on 13 July 2018. It has also secured European marketing authorization for treating Smallpox, Monkeypox, and Cowpox virus infections. Tecovirimat targets the viral protein p37, an essential component of the orthopoxvirus replication complex responsible for the maturation of the virus particles and their release from infected cells. By inhibiting the activity of p37, Tecovirimat effectively interferes with the viral replication cycle, preventing the spread of the virus within the host (Shiryaev et al., 2021; Sen Gupta et al., 2023).

Despite the genomics analysis of MPV, the proteome study is still challenging, and there is only a minimal solved protein structure, such as the highly conserved A42R protein, the first protein structure (resolution: 1.52 Å) deposited in the Protein Data Bank (PDB: 4QWO). Encoded by gp153 locus, the A42R protein role is still discussed despite the biochemical data suggesting its implication in replicating an assembly of orthopoxviruses. The current study’s findings and knowledge of its structure indicate that A42R could be a therapeutic target for structure-based drug design (Minasov et al., 2022; Bajrai et al., 2022).

The current study collected available natural molecules with validated chemical structures from the Eximed Laboratory. Using *in silico* approaches, they were evaluated as candidate inhibitors of the A42R Profilin-like Protein of Monkeypox Virus. The ADMET predictions for the selected Salsoline Derivatives, Genistein, a Semisynthetic Derivative of Kojic Acid, and Naringenin show promising results, justifying their potential for experimental validation, which represents the weakness of the present study.

## Materials end methods

### Ligand preparation

Available Naturel molecules with validated chemical structures were collected from Eximed Laboratory (https://eximedlab.com/) on 30 August 2022 (Alesawy et al., 2021) and subsequently prepared for docking calculations using the LigPrep utility available in the Maestro 11.5 edition of the Schrödinger Software program. This process utilized the OPLS3 force field and involved selecting ionization states for pH values 7.0 and 2.0. Furthermore, the ligands were allowed to generate a maximum of 32 stereoisomers (Mssillou et al., 2024).

### Protein preparation

The three-dimensional crystal structure of the A42R Profilin-like Protein from the Monkeypox Virus (PDB: 4QWO), with a resolution of 1.52 Å, was retrieved from the Protein Data Bank in PDB format (Preet et al., 2022). The structure was subjected to the preparation procedure using the Protein Preparation Wizard in Schrödinger-Maestro v11.5, which includes the removal of all water molecules, the conversion of selenomethionine residues to methionine, and the addition of hydrogen atoms to heavy atoms. Minimization was carried out employing the OPLS3 force field, with a maximum allowable heavy atom RMSD (root-mean-square-deviation) set at 0.30.

The grid box coordinates were defined as x: 1.26, y: 6.4, and z: 25.9. The box had a volumetric spacing of 20 × 20 × 20. To establish the connection between the ligand and the protein-based grid box, the ‘Extra Precision’ (XP) mode was utilized, and the results were evaluated using the XP Gscore (Mssillou et al., 2024).

Three distinct modes were used to evaluate the prospective ligands, including high throughput virtual screening (HTVS), standard precision (SP), and extra precision (XP). This screening protocol was structured to iteratively improve ligand placements, beginning with HTVS, followed by SP mode, and culminating in XP mode (Veeramachaneni et al., 2015). Furthermore, ligand refinement involved applying the properties of absorption, metabolism, distribution, and excretion (ADMET) parameters and adhering to Lipinski’s Rule of Five using the QikProp tool within the Schrödinger Software, specifically version 11.5 of Maestro (Chebaibi et al., 2024). This evaluation was conducted based on the physicochemical and pharmacokinetic characteristics of various molecules analyzed in our study. Key factors considered included molecular weight, the number of hydrogen bond donors and acceptors, total solvent-accessible surface area, the blood-brain partition coefficient, the octanol/water partition coefficient, and aqueous solubility (Kumar et al., 2023; Sharma et al., 2022).

A redocking method was implemented to assess the molecular docking protocol’s reliability. This entailed docking the co-crystallized ligand into its binding site. The precision and validity of the docking protocol were determined by achieving a root mean square deviation (RMSD) value below 2 Å between the initial and docked ligand poses (Beniwal et al., 2022).

### Molecular dynamics (MD) simulations

MD simulations were conducted utilizing GROMACS software (version 2019.3) to investigate the conformational dynamics of the most favorable docking complexes using the CHARMM 27 force field (Al-Khafaji and Taskin Tok, 2021). The protein topology was constructed using the GROMACS pdb2gmx modules with Chemistry at Harvard Macromolecular Mechanics force-field (CHARMm ff) (Chen et al., 2014), and the ligand topology was generated via the SwissParam server (Zoete et al., 2011). The docked structures were immersed in a simulation box with dimensions of 9.6 nm on each side and solvated using the three-point transferable intermolecular potential (TIP3P) solvent (Mark and Nilsson, 2001). To ensure system neutrality, ten chloride (Cl were introduced when needed. The system was then subjected to energy minimization using the steepest descent algorithm, with a maximum force threshold of 1,000 kJ/mol/nm. The pressure and temperature were then set to 1 bar and 300 K using the Nose-Hoover thermostat and isotropic Parrinello-Rahman barostat (Mahmoudi Gomari et al., 2022). Finally, a 100 ns simulation was carried out for each docked complex. We employed custom scripts derived from the results of molecular dynamics simulations to compute several metrics, such as root mean square deviation (RMSD) (utilizing ‘gmx rms’), root mean square fluctuation of residues (with ‘gmx rmsf’), Solvent Accessible Surface Area (SASA), the radius of gyration (Rg), and the count of hydrogen bonds (utilizing ‘gmx hbond').

### MM-PBSA calculation

The binding of free energies for the ligands (ΔEbind) was calculated using the Molecular Mechanics Poisson-Boltzmann Surface Area (MM-PBSA) method (Kumari et al., 2014). ΔEbind is calculated using the following equations:

$$\Delta \text{EMMPBSA}=\text{Ecomplex }‐\;\left(\text{Eprotein}+\text{Eligand}\right)$$ (1)

ΔGMMPBSA=ΔGvdw+ΔGele+ΔGpolar (2)

Equation 1: Ecomplex is the total MMPBSA energy of the protein-ligand complex. Eprotein and Eligand are the total solution-free energies of the isolated protein and ligand, respectively. Equation 2: MM-G/PBSA is the sum of electrostatic (Eele), van der Waals (EvdW), polar (Gpolar), and nonpolar (Gnonpolar) energies.

## Results and discussion

### Virtual screening results

**FIGURE 1:**
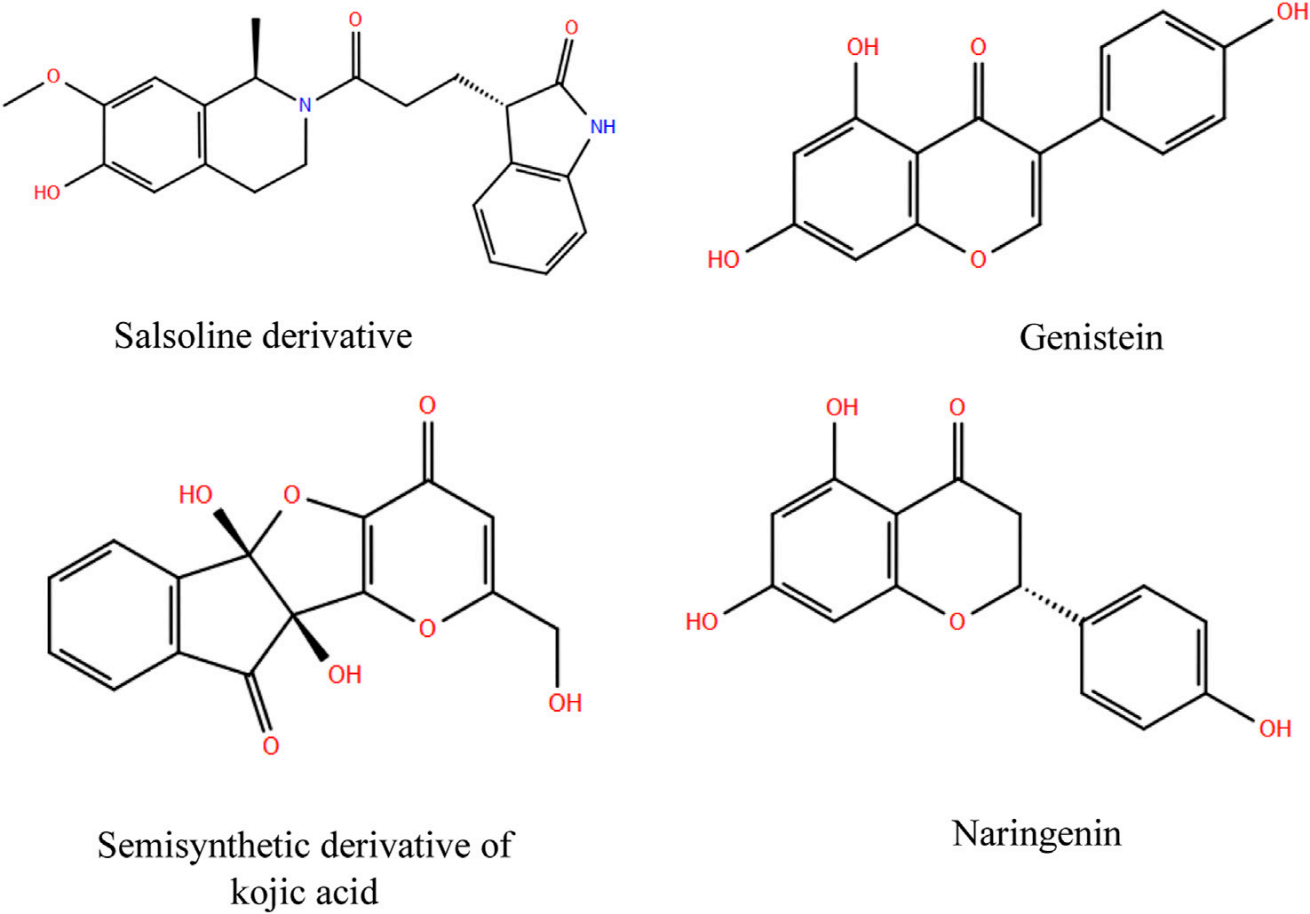
Selected natural products as potential MPV inhibitors.

Collected compounds were initially assessed for their inhibitory effects against MPV. Based on a combination of pharmacokinetic and pharmacological criteria, a subset of 6,360 molecules were selected for further analysis through High Throughput Virtual Screening (HTVS). Among these, a refined selection of 900 natural products underwent evaluation using Standard Precision (SP) and Extra Precision (XP), identifying 61 promising candidates (Supplemental Files). Based on the docking score, glide emodel score, and glide energy score, the Salsoline derivative, Genistein, Semisynthetic derivative of kojic acid, and Naringenin have been selected as candidate ligands to the active site of the A42R Profilin-like Protein from the MPV (PDB: 4QWO) (Figure 1).

Salsoline, a tetrahydroisoquinoline alkaloid extract from Salsola plants, is known for its diverse biological activities, including antibacterial effectiveness against *Staphylococcus aureus, Streptococcus mutans, Bacillus subtilis, Streptococcus pneumoniae* (Ou and Kwok, 2004; Wang et al., 2004; Wang et al., 2020) and antiviral activity against influenza A and B viruses (Wang et al., 2004). In the present *in silico* investigation, the Salsoline derivative exhibited the highest docking score, glide emodel score, and glide energy score, registering at −5.679, −49.575, and −36.546 kcal/mol, respectively (Table 1).

**TABLE 1:** Docking results with ligands in A42R Profilin-like Protein from MPV (PDB:4QWO).

Compound	Glide gscore (Kcal/mol)	Glide emodel (Kcal/mol)	Glide energy (Kcal/mol
Salsoline derivative	−5.679	−49.575	−36.546
Genistein	−5.617	−47.838	−33.885
Semisynthetic derivative of kojic acid	−5.585	−46.373	−33.428
Naringenin	−5.278	−40.661	−38.038
Tecovirimat	−3.477	−30.647	−25.482

Genistein, identified as a potential A42R inhibitor, displayed a docking score, glide emodel score, and glide energy score of −5.617, −47.838, and −33.885 kcal/mol, respectively (Table 1). This phytoestrogen isoflavone is abundantly found in sources like soy and dairy products. It manifests various biological actions such as antioxidant activity, vermifuge activity, DNA topoisomerase inhibition, and tyrosine-protein kinase inhibition (Dixon and Ferreira, 2002; Polkowski and Mazurek, 2000). Notably, it has demonstrated antiviral efficacy against several viruses, including HSV-1, cytomegalovirus, and human immunodeficiency virus (LeCher et al., 2019).

Naringenin, the fourth selected molecule, displayed a docking score, glide emodel score, and glide energy score of −5.278, −40.661, and −38.038 kcal/mol, respectively. As a flavonoid in the flavanone class, it is predominantly found in citrus fruits, grapefruit leaves, and celery seeds. Naringenin’s diverse activities encompass anti-atherogenic, anti-inflammatory, anti-mutagenic, anticancer, and antiviral effects (Yin et al., 2018; Karim et al., 2018; Ke et al., 2017; Pinho-Ribeiro et al., 2016; Salehi et al., 2019).

Genistein and naringenin, isomers of the well-known antimicrobial and antiviral isocoumarins, demonstrated significant inhibitory effects on the profilin-like protein A42R. The structural similarity between these flavonoids and isocoumarins may underlie their observed bioactivity, reinforcing the potential of these compounds as antiviral agents. Previous studies on isocoumarins have shown their broad-spectrum bioactivity, which could inform further exploration of genistein and naringenin against MPV proteins (Ramanan et al., 2016; Sudarshan et al., 2015; Sudarshan et al., 2019).

Various microorganisms form kojic acid from the aerobic degradation of carbohydrates. Kojic acid and its derivatives are known for their biological activities, including antimicrobial and antiviral (Aytemir and Özçelik, 2010), antitumor (Nawarak et al., 2008), antidiabetic (Xiong and Pirrung, 2008), and anticancer (Yoo et al., 2010) activities. Through our virtual screening, a semisynthetic derivative of kojic acid emerged as a potential MPV inhibitor, garnering a docking score, glide emodel score, and glide energy score of −5.585, −46.373, and −33.428 kcal/mol, respectively (Table 1).

In the present study, as a reference, the Tecovirimat was docked within the active site of the A42R and displayed a docking score, glide model score, and glide energy score of −3.477, −30.647, and −25.482 kcal/mol, respectively (Table 1). Compared to Tecovirimat, this result underscores the heightened effectiveness of the four selected natural molecules as a candidate inhibitor of A42R.

**FIGURE 2:**
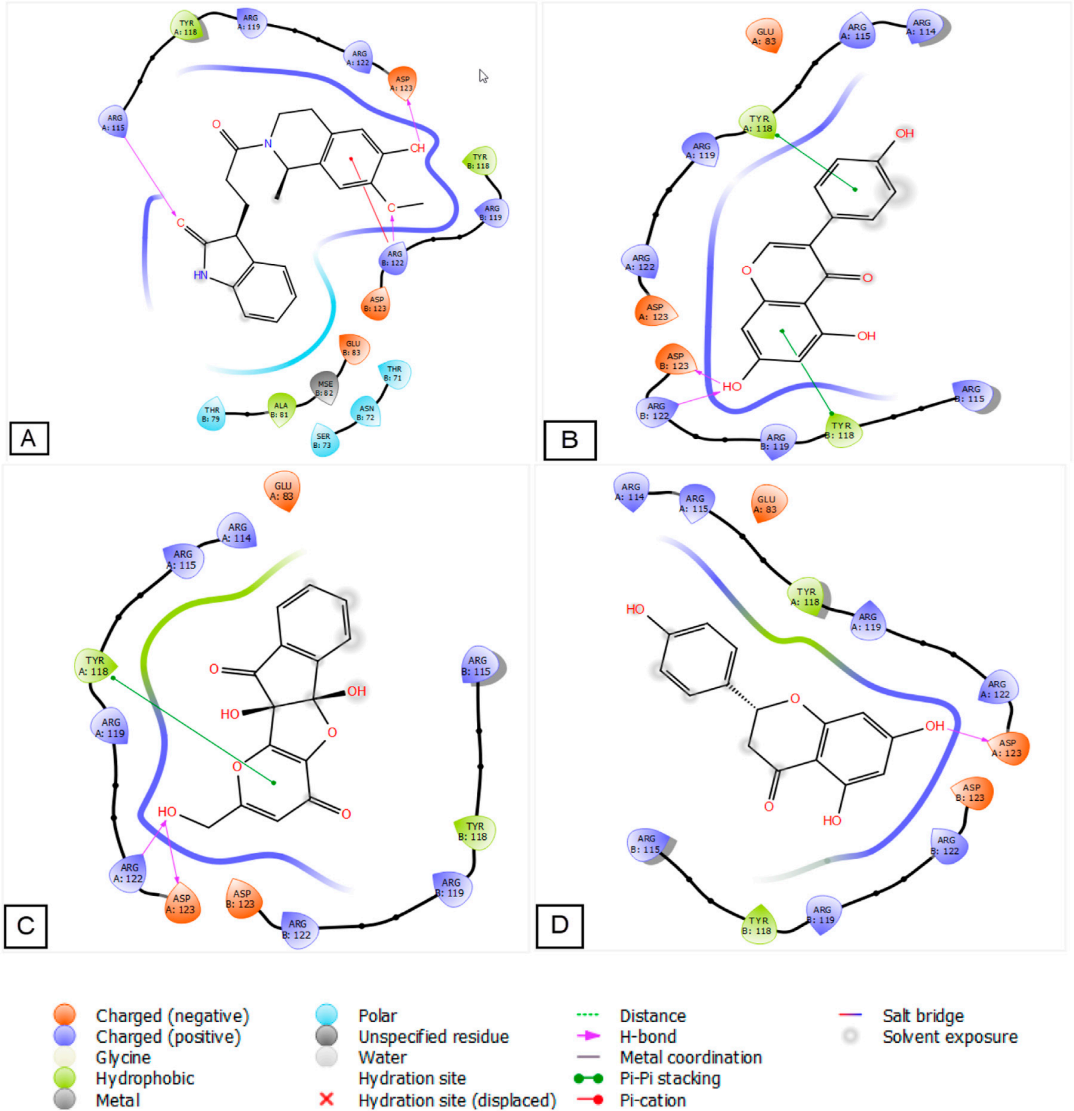
2D diagrams of candidate ligand’s interactions with the active sites. **(A)** Salsoline derivative; **(B)** Genistein; **(C)** Semisynthetic derivative of kojic acid; **(D)** Naringenin.

**FIGURE 3:**
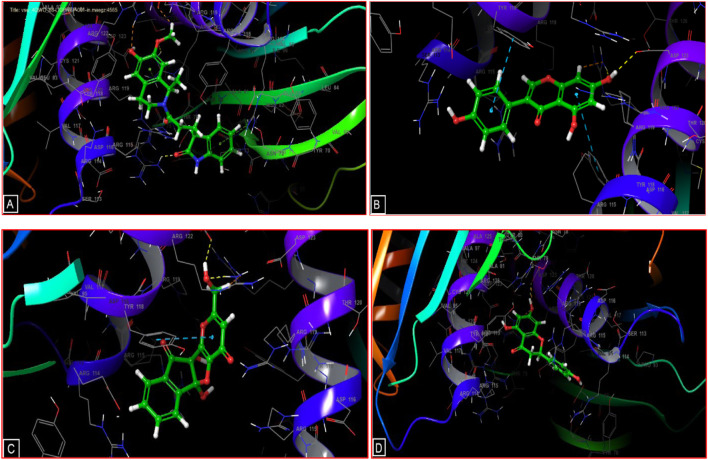
3D diagrams of candidate ligands interactions with the active sites. **(A)** Salsoline derivative; **(B)** Genistein; **(C)** Semisynthetic derivative of kojic acid; **(D)** Naringenin.

Figures 2, 3 illustrate the molecular docking interactions, revealing the nature and quantity of potential bonds formed between the ligands and the active site of the A42R. The Salsoline derivative exhibited notable interactions, establishing three hydrogen bonds with ARG 115, ARG 122, and ASP 123 residues and a Pi cation bond with residue ARG 122. Genistein established two hydrogen bonds with residues ARG 122 and ASP 123, intriguingly, 2 Pi-Pi stacking bonds with TYR A 118 and TYR B 118. The semisynthetic kojic acid derivative displayed two hydrogen bonds with ARG 122 and ASP 123 residues and a Pi-Pi stacking bond with TYR A 118. Naringenin established a single hydrogen bond with residue ASP 123 in its interactions. These interactions highlight the diverse bonding patterns contributing to the potential ligand binding and inhibitory effects against the A42R Profilin-like Protein.

Bioavailability hinges on the interplay between the active compound’s absorption, distribution, metabolism, and excretion (ADME) rates, with its physicochemical attributes playing a crucial role (El-Seedi et al., 2012). ADME predictions offer insights into the natural product characteristics and pharmacokinetic attributes of compounds identified as potential inhibitors for A42R. Interestingly, the selected four compounds exhibit a molecular mass below 500 Mol and possess favorable values (≤5 and ≤10, respectively) for hydrogen bond donors and acceptors (Table 2). Moreover, the oral bioavailability of drug compounds is significantly impacted by the magnitude of the solvent-exposed surface area (Khan et al., 2019). Interestingly, the Salsoline derivative, Genistein, the Semisynthetic derivative of kojic acid, and Naringenin demonstrate satisfactory values falling within the 300 to 1,000 range. Our study’s results agree with the predicted apparent Caco-2 cell permeability (QPP Caco), representing the gut-blood barrier (with values below 25 indicating poor permeability and those above 500 signifying excellent permeability). Each selected candidate inhibitor of A42R Profilin-like Protein had an acceptable QPP Caco value, ranging from 56.587 to 186.558 nm/s.

**TABLE 2:** ADME properties of natural products selected as potential MPV inhibitors.

Compound	MM^a^	Donors HB^b^	Acceptors HB^c^	SASA^d^	QPP caco^e^	QP logPo/w^f^	QPlogBB^g^	QP logS^h^	% human oral Absorption^i^
**Salsoline derivative**	380.443	2	7	673.712	186.558	2.309	−1.386	−4.443	81.11
**Genistein**	270.241	2	3.75	485.362	163.953	1.717	−1.335	−3.089	76.639
**Semisynthetic derivative of kojic acid**	302.24	3	8.45	505.571	56.587	−0.235	−1.797	−2.411	56.937
**Naringenin**	272.257	2	4	499.696	130.037	1.656	−1.398	−3.432	74.482

^**a**^ MM: mass of molecules (acceptable range: 500 mol).

^**b**^ Donors HB: donor of hydrogen bonds (acceptable range: ≤5).

^**c**^ Acceptors HB: acceptors of hydrogen bonds (acceptable range: ≤10).

^**d**^ SASA: Total solvent accessible surface area using a probe with a 1.4 radius (acceptable range: 300–1,000 radius).

^e^ QPP, Caco: Predicted apparent Caco-2, cell permeability in nm/s. Caco-2, cells is a model for the gut-blood barrier (˂25-poor, ˃500-great).

^**f**^ QP, logPo/w: Predicted octanol/water partition coefficient (acceptable range: −2–6.5).

^**g**^ QPlogBB: Predicted blood-brain partition coefficient (acceptable range: −3–1.2).

^**h**^ QP, logS: predicted aqueous solubility, S in mol/dm−3 (acceptable range: −6.5–0.5).

^**i**^ Predicted human oral absorption on 0%–100% scale (<25% is poor, and >80% is high).

Furthermore, these compounds exhibit advantageous blood-brain partition coefficient values, falling within the −3 to −1.2 range (as reported in reference (Pinho-Ribeiro et al., 2016)), suggesting their potential ability to cross the blood-brain barrier. As shown in Table 2, the anticipated oral absorption rates for these molecules range from 56% to 81%. Based on these promising results, the subsequent exploration aimed to delve into the molecular dynamics aspects of the four selected compounds.

#### Molecular dynamics (MD) simulation

**FIGURE 4:**
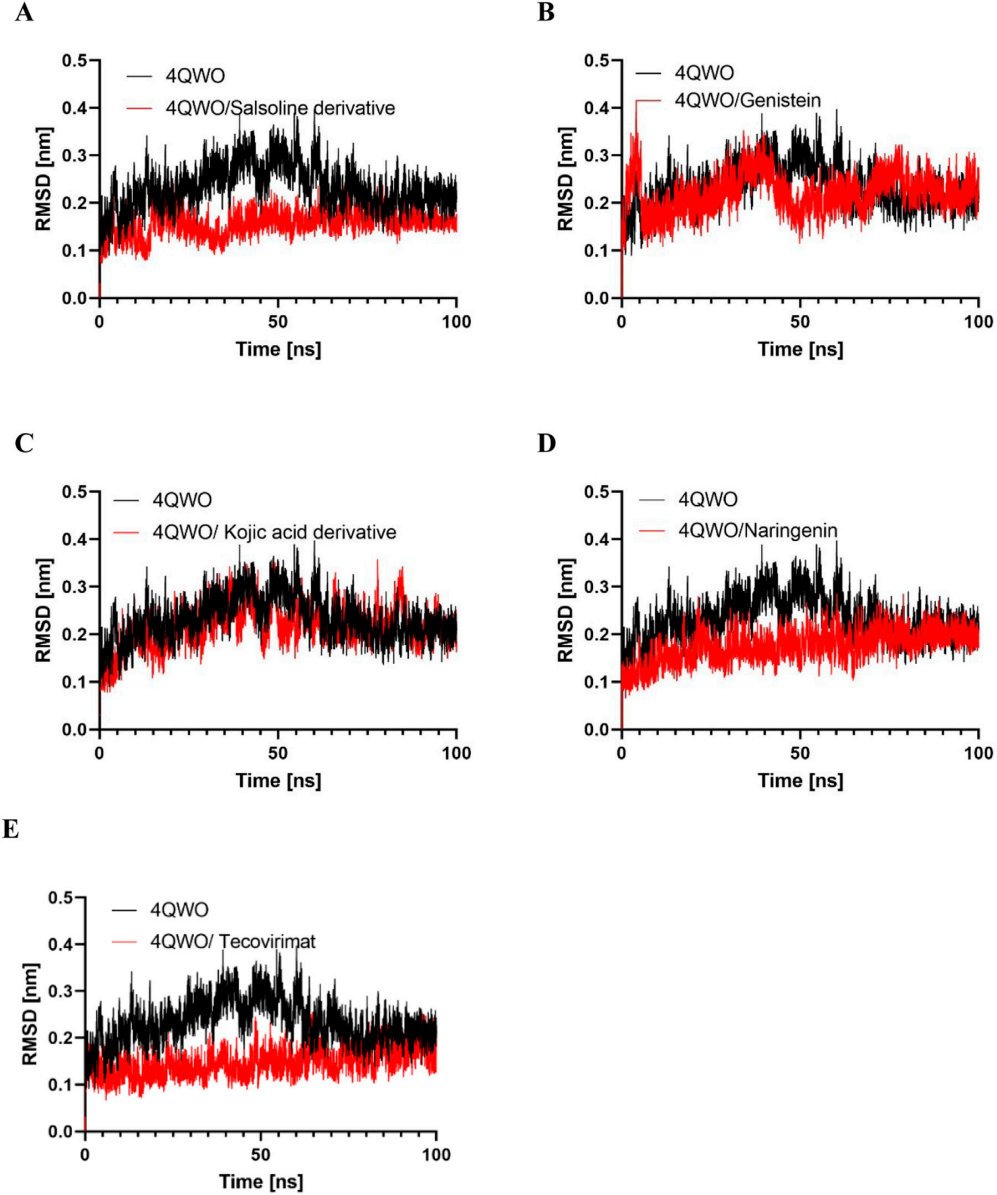
Root mean square deviation (RMSD) of protein backbone vs simulation time for solvated 4QWO protein free and complexed with **(A)** Salsoline derivative, **(B)** Genistein, **(C)** Semisynthetic derivative of kojic acid, **(D)** Naringenin, and **(E)** Tecovirimat during 100 ns of molecular dynamics simulations.

The application of MD simulation revealed new insights into the stability and dynamic nature of protein-ligand interactions, configured space of sampling, inter-atomic forces calculation, generation of the trajectory that is then followed by the dynamics of a protein’s interaction with its ligand during the molecular motions and effect at a specific time (Shukla and Tripathi, 2020). In this study, MD simulations have been applied to assess the structural stability of A42R (PDB: 4QWO), lasting 100 ns, both in its unbound (apo) and ligand-bound states. In addition, Root-Mean-Square-Deviation (RMSD) analysis (Belhassan et al., 2021; Ouassaf et al., 2021) has been used to assess the stability of these systems. The RMSD analysis revealed the following average values for the 4QWO protein in various states: 0.23 nm for the unbound form, 0.15 nm for the Salsoline derivative complex, 0.21 nm for the Genistein complex, 0.17 nm for the Semisynthetic derivative of kojic acid complex, 0.15 nm for the Naringenin complex, and 0.15 nm for the Tecovirimat complex (Table 3). The values of all three systems consistently established that the simulations remained stable throughout the 100 nanoseconds, providing a reliable measure of conformational changes over time. The backbone RMSD plot analysis revealed the complexes’ stability during the entire MD simulation despite a minor deviation from their initial conformations (Figure 4). The MD simulation results indicate that the RMSD of the protein backbone in the APO protein (without any compound) was higher than that of the Salsoline derivative, Genistein, Semisynthetic derivative of kojic acid, Naringenin, and Tecovirimat-protein complex structures which suggests less stability of the APO protein under physiological conditions. For the 4QWO/Genistein and 4QWO/Semisynthetic derivative of kojic acid complexes, the RMSD graphs exhibited an upward trend, with RMSD values increasing from 0.10 to 0.40 nm between 0 and 70 ns, indicating that the compounds were adjusting to a new conformation within the binding pocket. Subsequently, the RMSD values plateaued and stabilized at around 0.20 nm, not exceeding the 0.3 nm threshold. In contrast, the 4QWO/Salsoline derivative, 4QWO/Naringenin, and 4QWO/Tecovirimat complexes achieved stability at approximately 20 ns, and there was no significant deviation in the protein backbone atoms for the remainder of the simulation, resulting in a final RMSD values ranging from 0.20 to 0.18 nm.

**TABLE 3:** The calculated parameters for all the systems were obtained after 100 ns MD simulations.

Complex	Average RMSD (nm)	Average RMSF (nm)	Radius of gyration (nm)	Average SASA (backbone, nm^2^)
Apo 4QWO	0.23 ± 0.04	0.11 ± 0.04	2.04 ± 0.01	142.73 ± 1.36
4QWO - Salsoline derivative	0.15 ± 0.02	0.09 ± 0.04	2.01 ± 0.01	143.33 ± 1.28
4QWO - Genistein	0.22 ± 0.04	0.10 ± 0.03	2.00 ± 0.01	143.69 ± 1.25
4QWO - Semisynthetic derivative of kojic acid	0.21 ± 0.04	0.10 ± 0.04	2.00 ± 0.01	143.26 ± 1.26
4QWO - Naringenin	0.17 ± 0.03	0.10 ± 0.03	2.02 ± 0.01	143.10 ± 1.24
4QWO - Tecovirimat	0.15 ± 0.02	0.09 ± 0.03	2.01 ± 0.01	142.64 ± 1.26

Molecular Mechanics Poisson-Boltzmann Surface Area (MMPBSA) analysis.

#### Root mean square fluctuation (RMSF)

**FIGURE 5:**
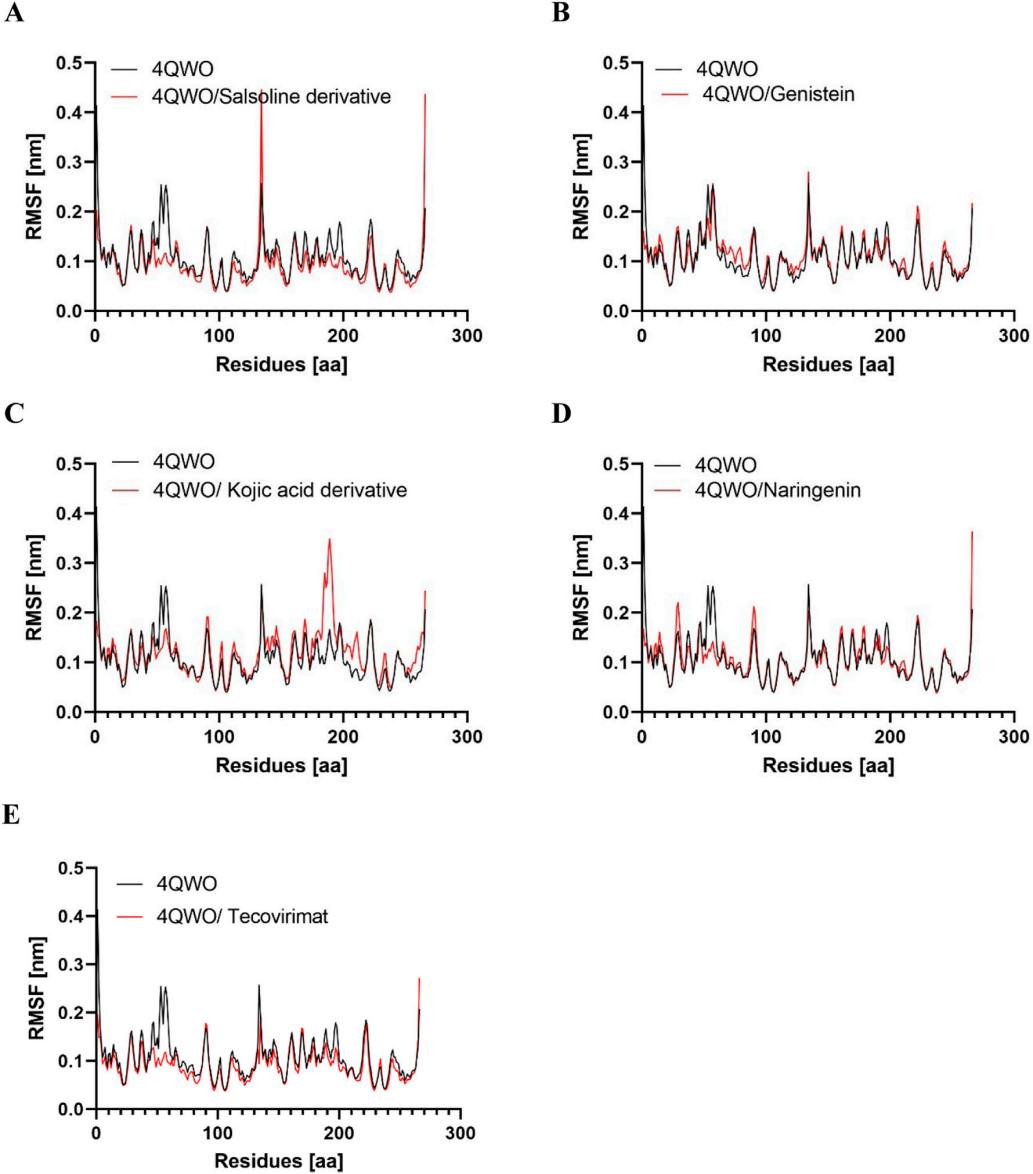
Root mean square deviation (RMSF) values of 4QWO alone and in complex with **(A)** Salsoline derivative, **(B)** Genistein, **(C)** Semisynthetic derivative of kojic acid, **(D)** Naringenin, and **(E)** Tecovirimat vs. the number of residues.

RMSF analysis was employed to explore the impact of ligand binding on the structural flexibility of the protein and the behavior of critical amino acids (Shao and Hall, 2017); a lower RMSF value suggests increased rigidity, while a higher value indicates greater flexibility (Khan et al., 2021). Figure 5 presents the RMSF profiles of Apo 4QWO, 4QWO - Salsoline derivative, Genistein, Semisynthetic derivative of kojic acid, Naringenin, and Tecovirimat complexes. A comparison to the Apo form of 4QWO reveals that the fluctuations in the residues of the ligand-bound complexes are stable, mainly in the ligand-binding regions. Additionally, the average RMSF values of 4QWO free, 4QWO - Salsoline derivative, Genistein, Semisynthetic derivative of kojic acid, Naringenin, and Tecovirimat complexes were 0.10, 0.09, 0.11, 0.10, and 0.09, respectively (Table 3), indicating that ligand binding has contributed to maintaining the structural stability of 4QWO.

#### Radius of gyration (Rg)

**FIGURE 6:**
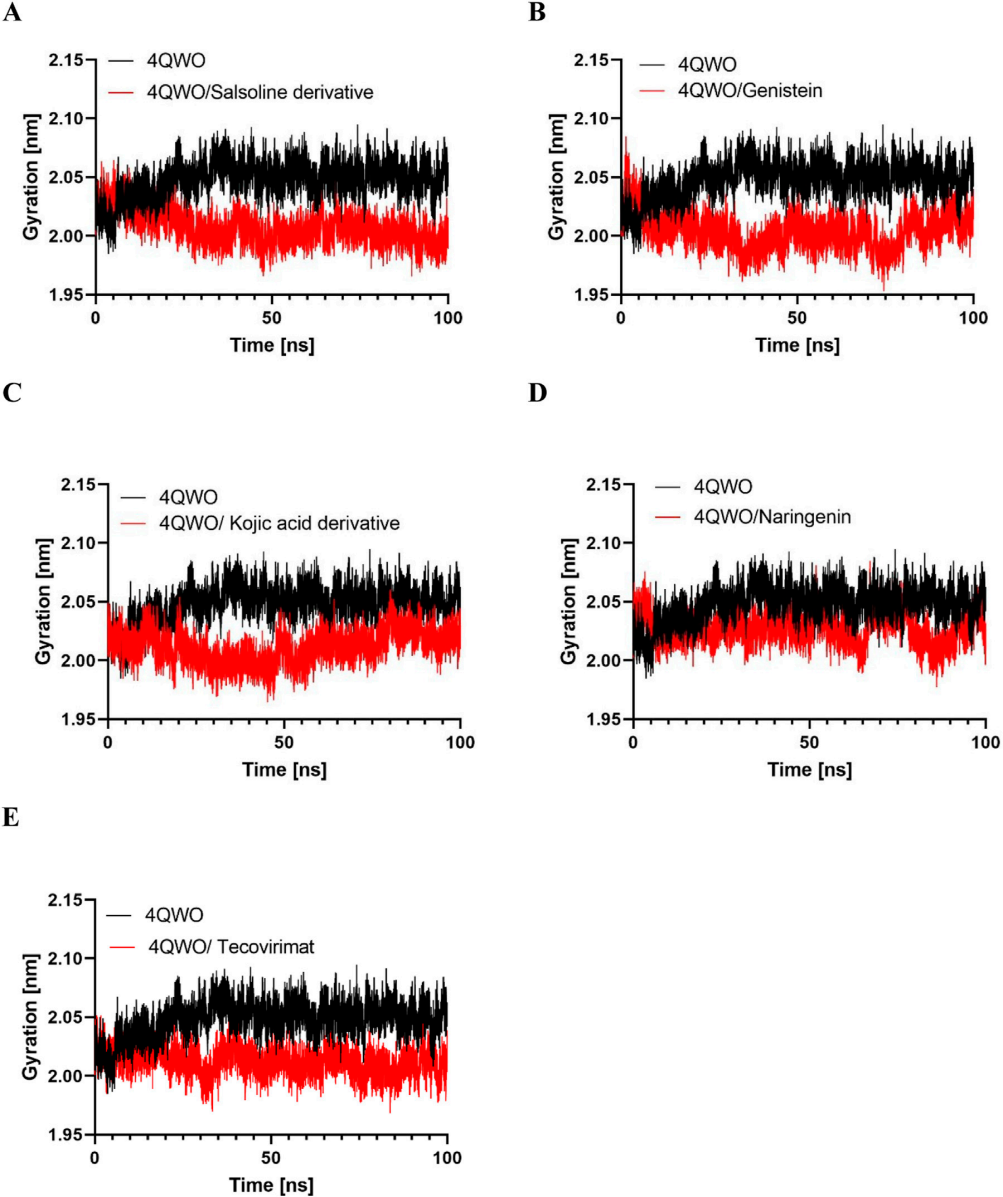
The radius of gyration (Rg) for backbone atoms of 4QWO alone and in complex with **(A)** Salsoline derivative, **(B)** Genistein, **(C)** Semisynthetic derivative of kojic acid, **(D)** Naringenin, and **(E)** Tecovirimat throughout the simulation.

To investigate the impact of various ligands on the overall compactness of the protein’s structure (El-Seedi et al., 2012), we examined the Rg over time; a ligand exhibiting a higher Rg value is more likely to display flexibility, indicating instability. Conversely, a lower Rg value suggests a denser and tightly packed conformation (Sharif et al., 2021). The average Rg values for Apo 4QWO, 4QWO-Salsoline derivative, Genistein, Semisynthetic derivative of kojic acid, Naringenin, and Tecovirimat complexes were determined to be 2.04, 2.01, 2.00, 2.00, 2.02, and 2.01, respectively (Table 3) which indicate that the binding to 4QWO did not induce a substantial alteration in compactness. Additionally, the graphical representation (Figure 6) illustrates that Rg values for the 4QWO - Tecovirimat, 4QWO - Salsoline derivative, and 4QWO - Semisynthetic derivative of kojic acid complexes achieved a stable equilibrium during the 100 ns simulation, in contrast to the 4QWO - Genistein and 4QWO - Naringenin complexes.

#### Solvent accessible surface area (SASA)

**FIGURE 7:**
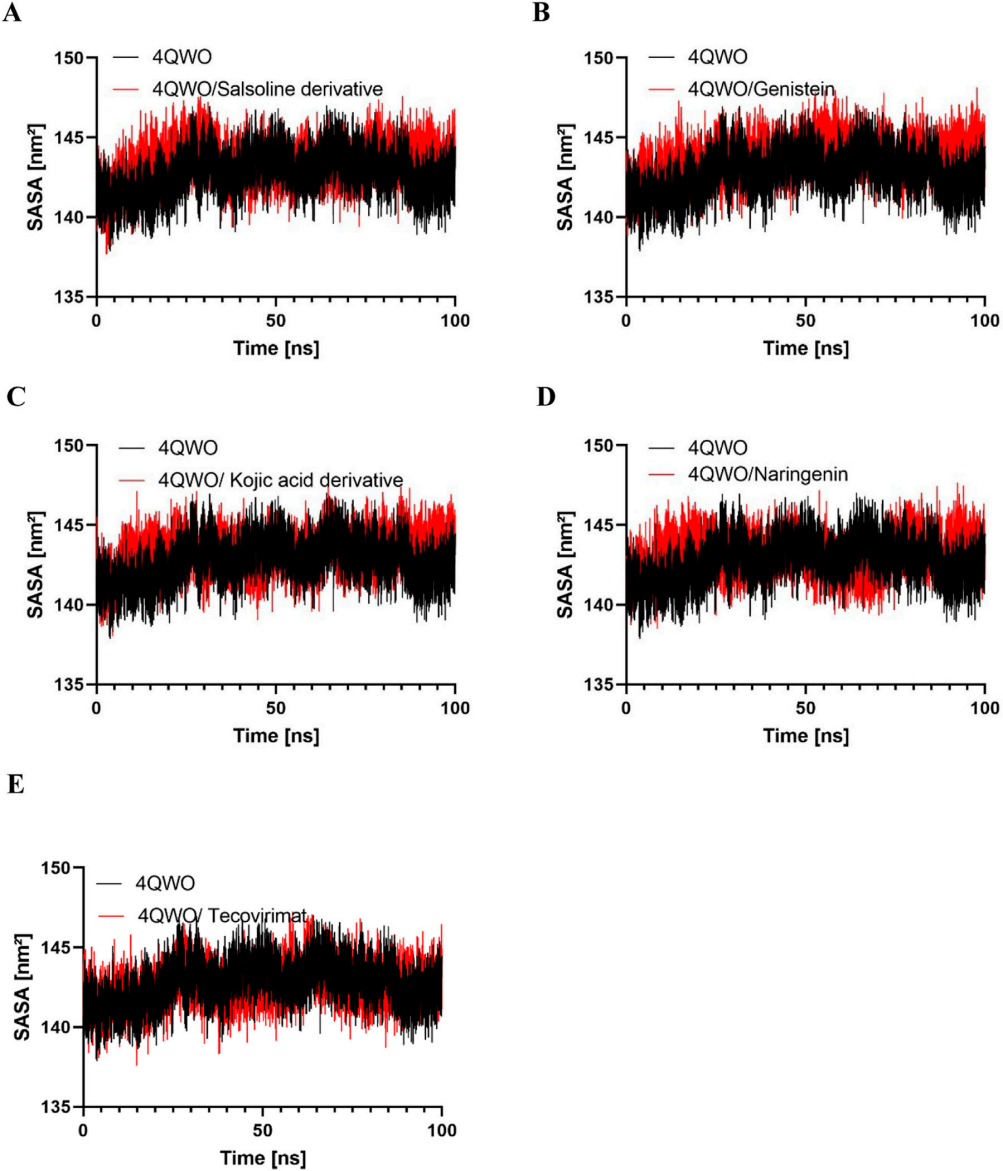
The comparative SASA values for backbone atoms of 4QWO alone and in complex with **(A)** Salsoline derivative, **(B)** Genistein, **(C)** Semisynthetic derivative of kojic acid, **(D)** Naringenin, and **(E)** Tecovirimat throughout the simulation.

SASA is defined as the area of a protein exposed to the surrounding solvent (Durham et al., 2009). The interpretation relies on assessing the connection between the surface of the macromolecule-ligand complex and the water molecules that are surrounded by (Priya et al., 2014). The variation in SASA for Apo 4QWO, 4QWO with a Salsoline derivative, Genistein, a Semisynthetic derivative of kojic acid, Naringenin, and Tecovirimat over a 100 ns period (Figure 7) showed an average value of 142.73 nm2, 143.33 nm2, 143.69 nm2, 143.26 nm2, 143.10 nm2, and 142.64 nm2, respectively (Table 3), which indicates that the ligands binding has no significant difference in the SASA values.

#### Hydrogen bonds analysis

**FIGURE 8:**
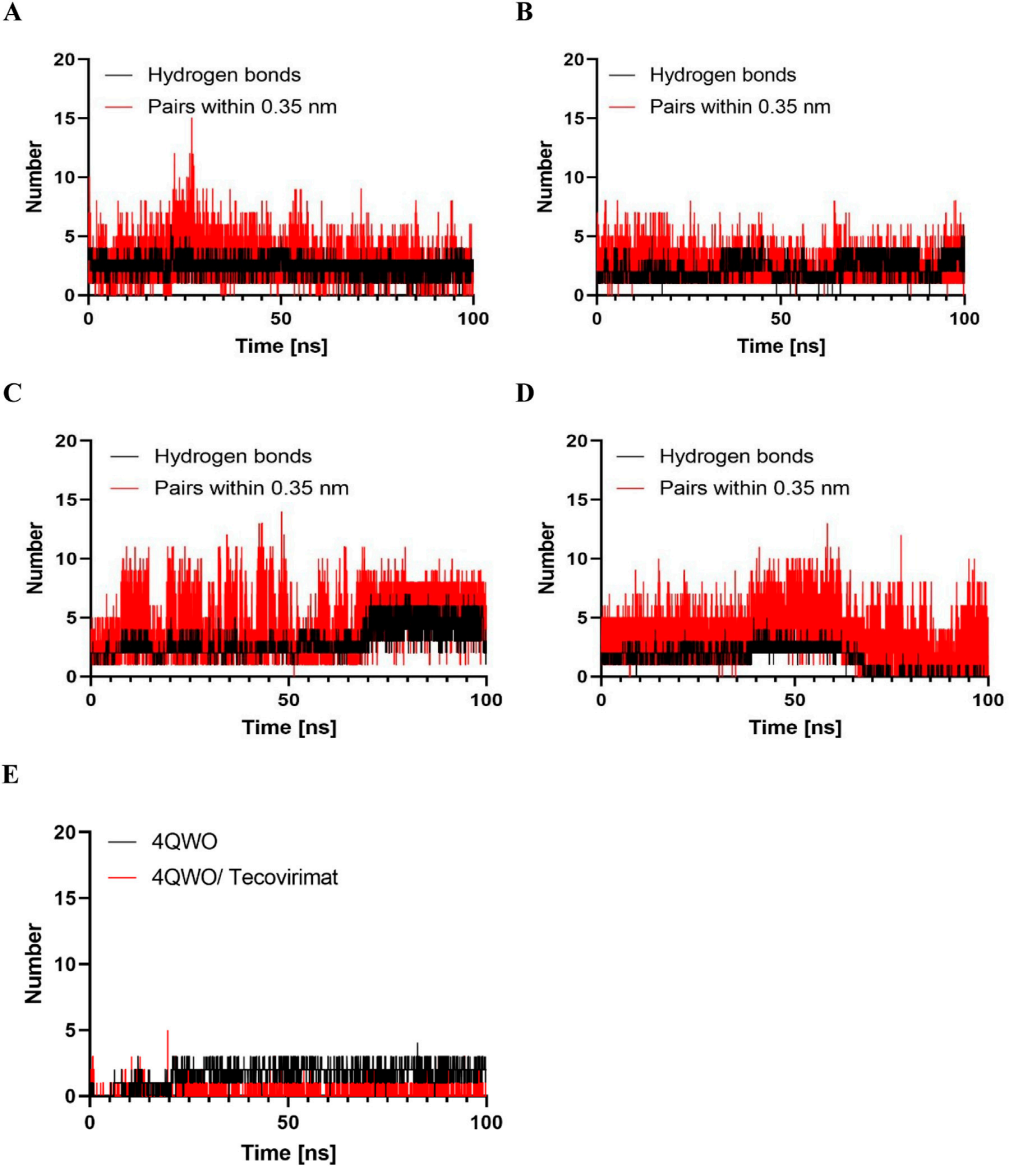
Hydrogen bond numbers made between **(A)** Salsoline derivative, **(B)** Genistein, **(C)** Semisynthetic derivative of kojic acid, **(D)** Naringenin, and **(E)** Tecovirimat in the4QWO protein active site residues during MD simulations.

Hydrogen bond analysis is used to comprehend how the examined molecules are recognized at the molecular level, their interactions, and selectivity within the receptors. (Bissantz et al., 2010). This analysis helped monitor the protein-ligand interactions that emerged during MD simulation based on secondary structural elements. During the MD trajectories of the complexes, we quantified the number of hydrogen bonds formed. Figure 8 illustrates the counts of hydrogen bonds and pairs formed within a 0.35 nm distance for the 4QWO-Salsoline derivative, 4QWO-Genistein, 4QWO-Semisynthetic derivative of kojic acid, 4QWO-Naringenin, and 4QWO-Tecovirimat complexes throughout the MD simulation. The results revealed that the Salsoline derivative established an average of 2.67 hydrogen bonds with the active pocket of 4QWO. Similarly, Genistein and the Semisynthetic derivative of kojic acid acted as ligands, interacting with 4QWO within the binding site with an average of 2.45 and 2.96 hydrogen bonds, respectively. However, the average number of hydrogen bonds for the 4QWO-Naringenin and 4QWO-Tecovirimat complexes was 1.47 and 1.70, respectively. The hydrogen bond analysis plot indicated that the Salsoline derivative maintained a more robust interaction with the binding pockets of 4QWO throughout the simulation compared to the Genistein, the Semisynthetic derivative of kojic acid, and the Naringenin (Figure 5).

#### Molecular Mechanics Poisson-Boltzmann Surface Area (MMPBSA) analysis

The MMPBSA analysis (Rostami et al., 2022) was used to evaluate the complexes’ binding affinities. All the trajectory snapshots were employed to calculate the primary forces governing the interactions between the protein and ligand. The total binding free energies (ΔEbind) for these complexes were computed in kJ/mol, and the results are presented in Table 4. The average binding free energy of the complexes, which include the Salsoline derivative, Genistein, Semisynthetic derivative of kojic acid, Naringenin, and Tecovirimat, were found to be −106.418, −46.808, −50.770, and −63.319 kJ/mol, respectively which are lower than −33.855 kJ/mol of the Tecovirimat complex. As indicated in Table 4, the Salsoline derivative exhibited the lowest binding energy, suggesting a weaker binding strength in this complex. This observation is consistent with the contributions of Van der Waals (VdW), electrostatic (Elec), and polar solvation energies to the binding energy, which align with the docking results.

**TABLE 4:** MM-PBSA calculations of binding free energy for All the complexes.

Complex	Binding energy (kJ/mol)	SASA energy (kJ/mol)	Polar solvation energy (kJ/mol)	Electrostatic energy (kJ/mol)	Van der waals energy (kJ/mol)
4QWO - Salsoline derivative	−106.418 + /- 58.464	−15.086 + /- 4.008	108.736 + /- 26.747	−67.391 + /- 25.660	−132.676 + /- 43.347
4QWO - Genistein	−46.808 + /- 35.016	−10.932 +/- 2.747	100.615 +/- 31.227	−51.243 +/- 38.730	−85.248 + /- 30.935
4QWO - Semisynthetic derivative of kojic acid	−50.770 + /- 24.495	−10.521 +/- 2.052	114.180 + /- 32.332	−71.260 + /- 31.135	−83.169 + /- 19.987
4QWO - Naringenin	−63.319 + /- 22.461	−12.709 + /- 1.899	95.126 + /- 28.289	−44.171 + /- 33.057	−101.565 + /- 19.561
4QWO - Tecovirimat	−33.855 + /- 28.604	−6.387 + /- 3.545	32.325 + /- 34.411	−7.179 + /- 11.858	−52.614 + /-31.400

## Conclusion

The recurrent spread of the Monkeypox virus has emerged as a global threat, posing significant health risks. Based on our virtual screening and molecular dynamics simulation, the Salsoline derivative, Genistein, the semi-synthetic derivative of kojic acid, and Naringenin have shown significant potential as inhibitors of the profilin-like protein A42R from the Monkeypox Zaire-96-I-16 virus (PDB: 4QWO).

Given that the absorption, distribution, metabolism, excretion, and toxicity (ADMET) predictions yielded promising results, we strongly recommend experimental validation to confirm the binding affinities of these compounds against A42R. This represents a limitation of the present study but also highlights an essential avenue for future research.

Looking forward, clinical trials to evaluate the anti-MPV activities of these natural compounds in patients infected with MPV are not only appropriate but also highly justified to further validate their therapeutic potential. Such studies could pave the way for developing new antiviral treatments, contributing significantly to public health efforts in managing and potentially curbing future outbreaks of the Monkeypox virus.

## Data Availability

The original contributions presented in the study are included in the article/Supplementary Material, further inquiries can be directed to the corresponding authors.

## References

[B1] Alesawy M. S. Elkaeed E. B. Alsfouk A. A. Metwaly A. M. Eissa I. H. (2021). *In silico* screening of semi-synthesized compounds as potential inhibitors for SARS-CoV-2 papain-like protease: pharmacophoric features, molecular docking, ADMET, toxicity and DFT studies. Molecules 26 (21), 6593. 10.3390/molecules26216593 34771004 PMC8588135

[B2] Al-Khafaji K. Taskin Tok T. (2021). Understanding the mechanism of amygdalin’s multifunctional anti-cancer action using computational approach. J. Biomol. Struct. Dyn. 39 (5), 1600–1610. 10.1080/07391102.2020.1736159 32107968

[B3] Angelo K. M. Petersen B. W. Hamer D. H. Schwartz E. Brunette G. (2019). Monkeypox transmission among international travellers—serious monkey business?. J. Travel Med. 26 (5), taz002. 10.1093/jtm/taz002 30657959 PMC6682460

[B4] Arita I. Henderson D. A. (1976). Monkeypox and whitepox viruses in west and central Africa. Bull. World Health Organ. 53 (4), 347–353. 186209 PMC2366520

[B5] Aytemir M. D. Özçelik B. (2010). A study of cytotoxicity of novel chlorokojic acid derivatives with their antimicrobial and antiviral activities. Eur. J. Med. Chem. 45 (9), 4089–4095. 10.1016/j.ejmech.2010.05.069 20591538

[B6] Bajrai L. H. Alharbi A. S. El-Day M. M. Bafaraj A. G. Dwivedi V. D. Azhar E. I. (2022). Identification of antiviral compounds against monkeypox virus profilin-like protein A42R from plantago lanceolata. Molecules 27 (22), 7718. 10.3390/molecules27227718 36431817 PMC9697570

[B7] Belhassan A. Zaki H. Chtita S. Alaqarbeh M. Alsakhen N. Benlyas M. (2021). Camphor, artemisinin and sumac phytochemicals as inhibitors against COVID-19: computational approach. Comput. Biol. Med. 136, 104758. 10.1016/j.compbiomed.2021.104758 34411900 PMC8354799

[B8] Beniwal M. Jain N. Jain S. Aggarwal N. (2022). Design, synthesis, anticancer evaluation and docking studies of novel 2-(1-isonicotinoyl-3-phenyl-1 H-pyrazol-4-yl)-3-phenylthiazolidin-4-one derivatives as Aurora-A kinase inhibitors. BMC Chem. 16 (1), 61. 10.1186/s13065-022-00852-8 35978438 PMC9382805

[B9] Bissantz C. Kuhn B. Stahl M. (2010). A medicinal chemist’s guide to molecular interactions. J. Med. Chem. 53 (14), 5061–5084. 10.1021/jm100112j 20345171 PMC2905122

[B10] Bunge E. M. Hoet B. Chen L. Lienert F. Weidenthaler H. Baer L. R. (2022). The changing epidemiology of human monkeypox—a potential threat? A systematic review. PLoS neglected Trop. Dis. 16 (2), e0010141. 10.1371/journal.pntd.0010141 PMC8870502 35148313

[B11] Chebaibi M. Mssillou I. Allali A. Bourhia M. Bousta D. Boschi Gonçalves R. F. (2024). Antiviral activities of compounds derived from medicinal plants against SARS-CoV-2 based on molecular docking of proteases. J. Biol. Biomed. Res. 1 (1), 10–30. 10.69998/j2br1

[B12] Chen K.-C. Sun M. F. Chen H. Y. Lee C. C. Chen C. Y. C. (2014). Potential smoothened inhibitor from traditional Chinese medicine against the disease of diabetes, obesity, and cancer. BioMed Res. Int. 2014, 1–12. 10.1155/2014/873010 PMC4127221 25136636

[B13] Dixon R. A. Ferreira D. (2002). Genistein. Phytochemistry 60 (3), 205–211. 10.1016/s0031-9422(02)00116-4 12031439

[B14] World Health Organization (2022a). US monkeypox outbreak: situation summary.

[B15] Durham E. Dorr B. Woetzel N. Staritzbichler R. Meiler J. (2009). Solvent accessible surface area approximations for rapid and accurate protein structure prediction. J. Mol. Model. 15 (9), 1093–1108. 10.1007/s00894-009-0454-9 19234730 PMC2712621

[B16] El-Seedi H. R. El-Said A. M. A. Khalifa S. A. M. Göransson U. Bohlin L. Borg-Karlson A. K. (2012). Biosynthesis, natural sources, dietary intake, pharmacokinetic properties, and biological activities of hydroxycinnamic acids. J. Agric. food Chem. 60 (44), 10877–10895. 10.1021/jf301807g 22931195

[B17] Gruber M. F. (2022). Current status of monkeypox vaccines. npj Vaccines 7 (1), 1–3. 10.1038/s41541-022-00527-4 35977979 PMC9385639

[B18] Heymann D. L. Szczeniowski M. Esteves K. (1998). Re-emergence of monkeypox in Africa: a review of the past six years. Br. Med. Bull. 54 (3), 693–702. 10.1093/oxfordjournals.bmb.a011720 10326294

[B19] Kabuga A. I. El Zowalaty M. E. (2019). A review of the monkeypox virus and a recent outbreak of skin rash disease in Nigeria. J. Med. Virology 91 (4), 533–540. 10.1002/jmv.25348 30357851

[B20] Karim N. Jia Z. Zheng X. Cui S. Chen W. (2018). A recent review of citrus flavanone naringenin on metabolic diseases and its potential sources for high yield-production. Trends Food Sci. and Technol. 79, 35–54. 10.1016/j.tifs.2018.06.012

[B21] Ke J. Y. Banh T. Hsiao Y. H. Cole R. M. Straka S. R. Yee L. D. (2017). Citrus flavonoid naringenin reduces mammary tumor cell viability, adipose mass, and adipose inflammation in obese ovariectomized mice. Mol. Nutr. and food Res. 61 (9), 1600934. 10.1002/mnfr.201600934 28370954

[B22] Khan M. F. Verma G. Akhtar W. Shaquiquzzaman M. Akhter M. Rizvi M. A. (2019). Pharmacophore modeling, 3D-QSAR, docking study and ADME prediction of acyl 1, 3, 4-thiadiazole amides and sulfonamides as antitubulin agents. Arabian J. Chem. 12 (8), 5000–5018. 10.1016/j.arabjc.2016.11.004

[B23] Khan S. A. Zia K. Ashraf S. Uddin R. Ul-Haq Z. (2021). Identification of chymotrypsin-like protease inhibitors of SARS-CoV-2 via integrated computational approach. J. Biomol. Struct. Dyn. 39 (7), 2607–2616. 10.1080/07391102.2020.1751298 32238094

[B24] Kugelman J. R. Johnston S. C. Mulembakani P. M. Kisalu N. Lee M. S. Koroleva G. (2014). Genomic variability of monkeypox virus among humans, Democratic Republic of the Congo. Emerg. Infect. Dis. 20 (2), 232–239. 10.3201/eid2002.130118 24457084 PMC3901482

[B25] Kumar H. Sharma A. Kumar D. Marwaha M. G. Dhanawat M. Aggarwal N. (2023). Synthesis, biological evaluation and *in silico* studies of some new analogues of 3, 5-disubstituted thiazolidin-2, 4-dione. Future Med. Chem. 15 (24), 2257–2268. 10.4155/fmc-2023-0237 37982252

[B26] Kumar N. Acharya A. Gendelman H. E. Byrareddy S. N. (2022). The 2022 outbreak and the pathobiology of the monkeypox virus. J. Autoimmun. 131, 102855. 10.1016/j.jaut.2022.102855 35760647 PMC9534147

[B27] Kumari R. Kumar R. Lynn A. (2014). g_mmpbsa⋅ A GROMACS tool for high-throughput MM-PBSA calculations. J. Chem. Inf. Model. 54 (7), 1951–1962. 10.1021/ci500020m 24850022

[B28] LeCher J. C. Diep N. Krug P. W. Hilliard J. K. (2019). Genistein has antiviral activity against herpes B virus and acts synergistically with antiviral treatments to reduce effective dose. Viruses 11 (6), 499. 10.3390/v11060499 31159175 PMC6630448

[B29] Likos A. M. Sammons S. A. Olson V. A. Frace A. M. Li Y. Olsen-Rasmussen M. (2005). A tale of two clades: monkeypox viruses. J. General Virology 86 (10), 2661–2672. 10.1099/vir.0.81215-0 16186219

[B30] Mahmoudi Gomari M. Rostami N. Omidi-Ardali H. Arab S. S. (2022). Insight into molecular characteristics of SARS-CoV-2 spike protein following D614G point mutation, a molecular dynamics study. J. Biomol. Struct. Dyn. 40 (12), 5634–5642. 10.1080/07391102.2021.1872418 33475020 PMC7832383

[B31] Marennikova S. Seluhina E. M. Mal'ceva N. N. Cimiskjan K. L. Macevic G. R. (1972). Isolation and properties of the causal agent of a new variola-like disease (monkeypox) in man. Bull. World Health Organ. 46 (5), 599–611. 4340219 PMC2480798

[B32] Mark P. Nilsson L. (2001). Structure and dynamics of the TIP3P, SPC, and SPC/E water models at 298 K. J. Phys. Chem. A 105 (43), 9954–9960. 10.1021/jp003020w

[B33] McCollum A. M. Damon I. K. (2014). Human monkeypox. Clin. Infect. Dis. 58 (2), 260–267. 10.1093/cid/cit703 24158414 PMC5895105

[B34] Meyer H. Perrichot M. Stemmler M. Emmerich P. Schmitz H. Varaine F. (2002). Outbreaks of disease suspected of being due to human monkeypox virus infection in the Democratic Republic of Congo in 2001. J. Clin. Microbiol. 40 (8), 2919–2921. 10.1128/jcm.40.8.2919-2921.2002 12149352 PMC120683

[B35] Minasov G. Inniss N. L. Shuvalova L. Anderson W. F. Satchell K. J. (2022). Structure of the Monkeypox profilin-like protein A42Rreveals potential function differences from cellular profilins. bioRxiv, 2022. 10.1107/S2053230X22009128 PMC9527652 36189721

[B36] Mssillou I. Agour A. Lefrioui Y. Chebaibi M. (2024). LC-TOFMS analysis, *in vitro* and *in silico* antioxidant activity on NADPH oxidase, and toxicity assessment of an extract mixture based on Marrubium vulgare L. and Dittrichia viscosa L. J. Biol. Biomed. Res. 1 (1), 31–45. 10.69998/j2br2

[B37] Nawarak J. Huang-Liu R. Kao S. H. Liao H. H. Sinchaikul S. Chen S. T. (2008). Proteomics analysis of kojic acid treated A375 human malignant melanoma cells. J. Proteome Res. 7 (9), 3737–3746. 10.1021/pr7008737 18630942

[B38] Nuzzo J. B. Borio L. L. Gostin L. O. (2022). The WHO declaration of monkeypox as a global public health emergency. Jama 328, 615. 10.1001/jama.2022.12513 35895041

[B39] Ou S. Kwok K. C. (2004). Ferulic acid: pharmaceutical functions, preparation and applications in foods. J. Sci. Food Agric. 84 (11), 1261–1269. 10.1002/jsfa.1873

[B40] Ouassaf M. Belaidi S. Chtita S. Lanez T. Abul Qais F. Md Amiruddin H. (2021). Combined molecular docking and dynamics simulations studies of natural compounds as potent inhibitors against SARS-CoV-2 main protease. J. Biomol. Struct. Dyn. 40, 11264–11273. 10.1080/07391102.2021.1957712 34315340

[B41] Pinho-Ribeiro F. A. Zarpelon A. C. Fattori V. Manchope M. F. Mizokami S. S. Casagrande R. (2016). Naringenin reduces inflammatory pain in mice. Neuropharmacology 105, 508–519. 10.1016/j.neuropharm.2016.02.019 26907804

[B42] Polkowski K. Mazurek A. P. (2000). Biological properties of genistein. A review of *in vitro* and *in vivo* data. Acta Pol. Pharm. 57 (2), 135–155. 10934794

[B43] Preet G. Oluwabusola E. T. Milne B. F. Ebel R. Jaspars M. (2022). Computational repurposing of mitoxantrone-related structures against monkeypox virus: a molecular docking and 3D pharmacophore study. Int. J. Mol. Sci. 23 (22), 14287. 10.3390/ijms232214287 36430762 PMC9695275

[B44] Priya G. Doss C. Nagasundaram N. (2014). Molecular docking and molecular dynamics study on the effect of ERCC1 deleterious polymorphisms in ERCC1-XPF heterodimer. Appl. Biochem. Biotechnol. 172 (3), 1265–1281. 10.1007/s12010-013-0592-5 24158589

[B45] Ramanan M. Sinha S. Sudarshan K. Aidhen I. S. Doble M. (2016). Inhibition of the enzymes in the leukotriene and prostaglandin pathways in inflammation by 3-aryl isocoumarins. Eur. J. Med. Chem. 124, 428–434. 10.1016/j.ejmech.2016.08.066 27597418

[B46] Reynolds M. G. Yorita K. Kuehnert M. Davidson W. Huhn G. Holman R. (2006). Clinical manifestations of human monkeypox influenced by route of infection. J. Infect. Dis. 194 (6), 773–780. 10.1086/505880 16941343

[B47] Rizk J. G. Lippi G. Henry B. M. Forthal D. N. Rizk Y. (2022). Prevention and treatment of monkeypox. Drugs 82, 957–963. 10.1007/s40265-022-01742-y 35763248 PMC9244487

[B48] Rostami N. Choupani E. Hernandez Y. Arab S. S. Jazayeri S. M. Gomari M. M. (2022). SARS‐CoV‐2 spike evolutionary behaviors; simulation of N501Y mutation outcomes in terms of immunogenicity and structural characteristic. J. Cell. Biochem. 123 (2), 417–430. 10.1002/jcb.30181 34783057 PMC8657535

[B49] Salehi B. Fokou P. V. T. Sharifi-Rad M. Zucca P. Pezzani R. Martins N. (2019). The therapeutic potential of naringenin: a review of clinical trials. Pharmaceuticals 12 (1), 11. 10.3390/ph12010011 30634637 PMC6469163

[B50] Sen Gupta P. S. Panda S. K. Nayak A. K. Rana M. K. (2023). Identification and investigation of a cryptic binding pocket of the P37 envelope protein of monkeypox virus by molecular dynamics simulations. J. Phys. Chem. Lett. 14 (13), 3230–3235. 10.1021/acs.jpclett.3c00087 36972468

[B51] Shao Q. Hall C. K. (2017). Allosteric effects of gold nanoparticles on human serum albumin. Nanoscale 9 (1), 380–390. 10.1039/c6nr07665c 27924337 PMC5314867

[B52] Sharif M. Hossen N. L. Shaikat M. M. Haidary F. Ema T. I. Dey D. (2021). Molecular optimization, docking and dynamic simulationstudy of selective natural aromatic components to block E2-CD81 complex formation inpredating protease inhibitor resistant HCV influx. Int. J. Pharm. Res. 13 (2), 3511–3525. 10.31838/ijpr/2021.13.02.408

[B53] Sharma R. K. Bibi S. Chopra H. Khan M. S. Aggarwal N. Singh I. (2022). *In silico* and *in vitro* screening constituents of eclipta alba leaf extract to reveal antimicrobial potential. Evid. Based Complementary Altern. Med. 2022 (1), 1–14. 10.1155/2022/3290790 PMC9402321 36034950

[B54] Sherwat A. Brooks J. T. Birnkrant D. Kim P. (2022). Tecovirimat and the treatment of monkeypox—past, present, and future considerations. N. Engl. J. Med. 387, 579–581. 10.1056/nejmp2210125 35921403

[B55] Shiryaev V. A. Skomorohov M. Y. Leonova M. V. Bormotov N. I. Serova O. A. Shishkina L. N. (2021). Adamantane derivatives as potential inhibitors of p37 major envelope protein and poxvirus reproduction. Design, synthesis and antiviral activity. Eur. J. Med. Chem. 221, 113485. 10.1016/j.ejmech.2021.113485 33965861 PMC9533879

[B56] Shukla R. Tripathi T. (2020). “Molecular dynamics simulation of protein and protein–ligand complexes,” in Computer-aided drug design (Springer), 133–161.

[B57] Sudarshan K. Boda A. k. Dogra S. Bose I. Yadav P. N. Aidhen I. S. (2019). Discovery of an isocoumarin analogue that modulates neuronal functions via neurotrophin receptor TrkB. Bioorg. and Med. Chem. Lett. 29 (4), 585–590. 10.1016/j.bmcl.2018.12.057 30600206

[B58] Sudarshan K. Manna M. K. Aidhen I. S. (2015). Synthesis of 3‐arylisocoumarins by using acyl anion Chemistry and synthesis of thunberginol A and cajanolactone A. Eur. J. Org. Chem. 2015 (8), 1797–1803. 10.1002/ejoc.201403524

[B59] Tesh R. B. Watts D. M. Sbrana E. Siirin M. Popov V. L. Xiao S. Y. (2004). Experimental infection of ground squirrels (*Spermophilus tridecemlineatus*) with monkeypox virus. Emerg. Infect. Dis. 10 (9), 1563–1567. 10.3201/eid1009.040310 15498157 PMC3320280

[B60] Veeramachaneni G. K. kodamala K. R. Chalasani L. M. J S B. Talluri V. R. (2015). High-throughput virtual screening with e-pharmacophore and molecular simulations study in the designing of pancreatic lipase inhibitors. Drug Des. Dev. Ther. 9, 4397–4412. 10.2147/dddt.s84052 PMC4532172 26273199

[B61] Velavan T. P. Meyer C. G. (2022). Monkeypox 2022 outbreak: an update. Trop. Med. and Int. Health 27, 604–605. 10.1111/tmi.13785 35633308

[B62] Wang R. F. Feng Wang R. Wei Yang X. Mei Ma C. Nong Li J. Shoyama Y. (2004). A bioactive alkaloid from the flowers of Trollius chinensis. Heterocycles-Sendai Inst. Heterocycl. Chem. 63 (6), 1443–1448. 10.3987/com-04-10062

[B63] Wang S. Yan M. Guo Y. Sun R. Jin H. Gong Y. (2020). *In vivo* and *in vitro* effects of Salsola collina on gastrointestinal motility in rats. Iran. J. Basic Med. Sci. 23 (3), 383–389. 10.22038/IJBMS.2019.40613.9605 32440326 PMC7229513

[B64] World health organization *.* MonkeyPox, 2022b.

[B65] Xiong X. Pirrung M. C. (2008). Modular synthesis of candidate indole-based insulin mimics by Claisen rearrangement. Org. Lett. 10 (6), 1151–1154. 10.1021/ol800058d 18303898

[B66] Yin J. Liang Y. Wang D. Yan Z. Yin H. Wu D. (2018). Naringenin induces laxative effects by upregulating the expression levels of c-Kit and SCF, as well as those of aquaporin 3 in mice with loperamide-induced constipation. Int. J. Mol. Med. 41 (2), 649–658. 10.3892/ijmm.2017.3301 29207043 PMC5752176

[B67] Yoo D. S. Lee J. Choi S. S. Rho H. S. Cho D. H. Shin W. C. (2010). A modulatory effect of novel kojic acid derivatives on cancer cell proliferation and macrophage activation. Die Pharmazie-An Int. J. Pharm. Sci. 65 (4), 261–266. 10.1691/ph.2010.9764 20432622

[B68] Zoete V. Cuendet M. A. Grosdidier A. Michielin O. (2011). SwissParam: a fast force field generation tool for small organic molecules. J. Comput. Chem. 32 (11), 2359–2368. 10.1002/jcc.21816 21541964

